# Soil quality, elemental stoichiometry and crop yield under partial substitution of chemical fertilizers with organic inputs in Vertisols: a six-site field study

**DOI:** 10.3389/fpls.2025.1742932

**Published:** 2026-01-06

**Authors:** Fahui Jiang, Shang Han, Wenlong Cheng, Li Song, Shan Tang, Hui Wang, Rongyan Bu, Min Li, Rui Zhu, Mahbub Ul Islam, Ji Wu

**Affiliations:** 1 Soil and Fertilizer Institute, Anhui Academy of Agricultural Sciences (National Agricultural Experimental Station for Soil Quality, Taihe)/Anhui Provincial Key Laboratory of Nutrient Cycling and Arable Land Conservation, Hefei, China; 2 Anhui Soil and Fertilizer Station, Hefei, China; 3 Bangladesh Agricultural Research Institute, Gazipur, Bangladesh

**Keywords:** nutrient availability, organic substitution, soil organic carbon, soil quality index, stoichiometric ratio

## Abstract

Partial substitution of chemical fertilizers with organic amendments represents a promising approach to sustainable soil management, yet its integrated effects on soil quality and elemental stoichiometry in Vertisols remain insufficiently understood. This study aimed to investigate the impacts of partial organic fertilizer substitution on soil quality, ecological stoichiometry, crop yield, and yield stability through a three-year, six-site field experiment in the North China Plain. Treatments included an unfertilized control, full chemical fertilization (NPK), and NPK partially substituted by pig manure at 15% and 30% of nitrogen input (15%M and 30%M). Results revealed that partial substitution significantly reduced soil bulk density (1.3–17.1%) and increased soil organic carbon (0.77–22.5%) compared with NPK and control plots. While total nitrogen (N), phosphorus (P), and potassium (K) contents were comparable to NPK, nutrient availability improved markedly (2.1–23.7%). Organic inputs also modified soil elemental stoichiometry by increasing C:N, C:P, and N:P ratios, indicating a shift toward more balanced nutrient states. These improvements translated into substantial increases in the Soil Quality Index (31.5–335.6%), which in turn supported significant yield gains in wheat and maize (1.3–251.3%), with the 30%M treatment consistently achieving the greatest benefits. Random forest analysis and structural equation modeling demonstrated that yield responses were predominantly mediated through improvements in soil quality, driven by enhanced nutrient availability and optimized stoichiometry. Overall, our findings suggest that partial substitution of chemical fertilizers with organic inputs-particularly at a 30% replacement rate-offers as an effective strategy to improve soil quality, mitigate nutrient imbalances, and promoting sustainable intensification of wheat–maize systems in Vertisol regions.

## 1 Introduction

Ensuring global food security under the constraints of population growth, shrinking arable land, and climate change represents a major challenge for sustainable agriculture (Godfray et al., 2010). The development of chemical fertilizers (CF) has been pivotal in supporting food production, contributing to approximately 50% of yield increases and sustaining nearly half of the global population (FAOSTAT, 2020). However, in recent decades, inefficient and excessive CF application has generated numerous adverse consequences, including soil acidification, water eutrophication, human health risks, and high resource consumption (Liu et al., 2021; Ren et al., 2021). Thus, strategies to reduce CF dependency while sustaining crop productivity are urgently needed.

Integrating organic amendments into fertilization regimes has been widely proposed as a sustainable alternative. Manure application can enhance nutrient supply, increase soil organic matter, and reduce reliance on CF. However, the sole use of organic fertilizers may constrain yields, highlighting the need for partial substitution strategies that balance nutrient availability, and soil fertility. Previous studies have reported that partial organic substitution increases soil organic matter by 8.96% - 15.9% in both short- and long-term trials (Cheng et al., 2017; Yang et al., 2020), and enhances soil total and available N, P, and K relative to unfertilized soils (Cheng et al., 2017; Gosal et al., 2018). These improvements are attributed to the addition of organic matter, which stimulates macro-aggregate formation and promotes iron oxide activation (Ren et al., 2024). Other reported benefits include enhanced soil water-holding capacity (Obour et al., 2018), improved wheat nutrient uptake, increased microbial diversity, and more complex microbial networks (He et al., 2024). Moreover, partial organic substitution can strengthen microbial community structures and functions, thereby improving nutrient availability and utilization efficiency (Yang et al., 2025). Importantly, this practice has also been shown to mitigate environmental risks, with reduced N_2_O emissions reduced by 11–78% when 20–80% of CF inputs are substituted by organic amendments (Li et al., 2021b). Collectively, these findings suggest that partial organic substitution can improve soil structure, enhance nutrient availability, promote soil health, increase biodiversity, and reduce environmental pollution. Although these studies demonstrate the benefits of partial organic substitution for soil fertility and environmental protection (Liu et al., 2023a, 2023b), comprehensive quantitative assessments of its effects on soil quality and soil elemental stoichiometry remain limited.

Soil quality is a multidimensional concept encompassing physical, chemical, and biological properties that together define soil function. While numerous single indicators (e.g., bulk density, pH, water-holding capacity, cation exchange capacity, microbial biomass) are often used to assess soil health, each reflects only a narrow aspect of soil functioning, and many properties are interdependent and context-dependent (Zhang et al., 2019). To overcome this limitation, the soil quality index (SQI), based on the minimum data set (MDS) approach, has been developed. SQI integrates multiple representative soil indicators, thereby reducing redundancy, minimizing multicollinearity, lowering measurement costs, improving efficiency, and enhancing comparability across regions (Rinot et al., 2019; Li et al., 2021a).

In addition to soil quality, soil elemental stoichiometry is increasingly recognized as a critical determinant of soil fertility and ecosystem functioning. Fertilization regimes alter stoichiometric relationships through shifts in nutrient inputs, microbial activity, and biogeochemical cycling (Abrar et al., 2020; Morón-Cruz et al., 2024). Elemental ratios such as C:N, C:P, and N:P act as regulatory “balancers” in farmland ecosystems, influencing soil structure, nutrient cycling, microbial activity, and plant productivity (Cui et al., 2018). Balanced stoichiometry promotes soil quality and fertility, whereas imbalances can destabilize soil-plant systems and trigger cascading ecological effects (Luo et al., 2025). Despite these insights, few studies have systematically examined the effects of partial organic substitution on soil elemental stoichiometry and its linkage to soil biogeochemistry, nutrient availability, microbial processes, and crop yields.

Vertisols (locally referred to as Shajiang Black Soils) occupy approximately 4 × 10^6^ ha in the southern North China Plain and constitute one of the region’s dominant soil types for crop production (Li, 2011). Although they play a critical role in regional food security, Vertisols are generally classified as medium- to low-yielding soils (Li, 2011). This low productivity arises from multiple unfavorable characteristics including weak structural stability, low soil organic carbon, limited nutrient availability, and consequently unstable crop yields (Chen et al., 2020; Wang et al., 2021; Ding et al., 2025). The predominance of montmorillonite confers a heavy texture, high shrink-swell potential, and poor tilth, restricting root development and complicating field management (Xiong et al., 2020; Chen et al., 2024). In addition, low quality organic matter (typically 10–15 g kg^−1^) further constrains aggregate formation and nutrient retention (Chen et al., 2020). Adverse edaphic conditions, such as waterlogging, drought susceptibility, and physiological hardening, exacerbate these limitations and collectively suppress soil quality, fertility, and crop productivity (Guo et al., 2021).

To address these challenges, we conducted a three-year, multi-site field experiment across six locations in the North China Plain to comprehensively evaluate the impacts of partial organic substitution in Vertisols. The specific objectives were to: (1) quantify the responses of soil structural and nutrient properties to partial substitution of CF with organic amendments; (2) assess its impacts on the soil quality index (SQI) and elemental stoichiometry; and (3) identify the key drivers and mechanisms mediating yield responses of wheat and maize under Vertisol conditions.

## 2 Materials and methods

### 2.1 Experimental site

**Figure 1:**
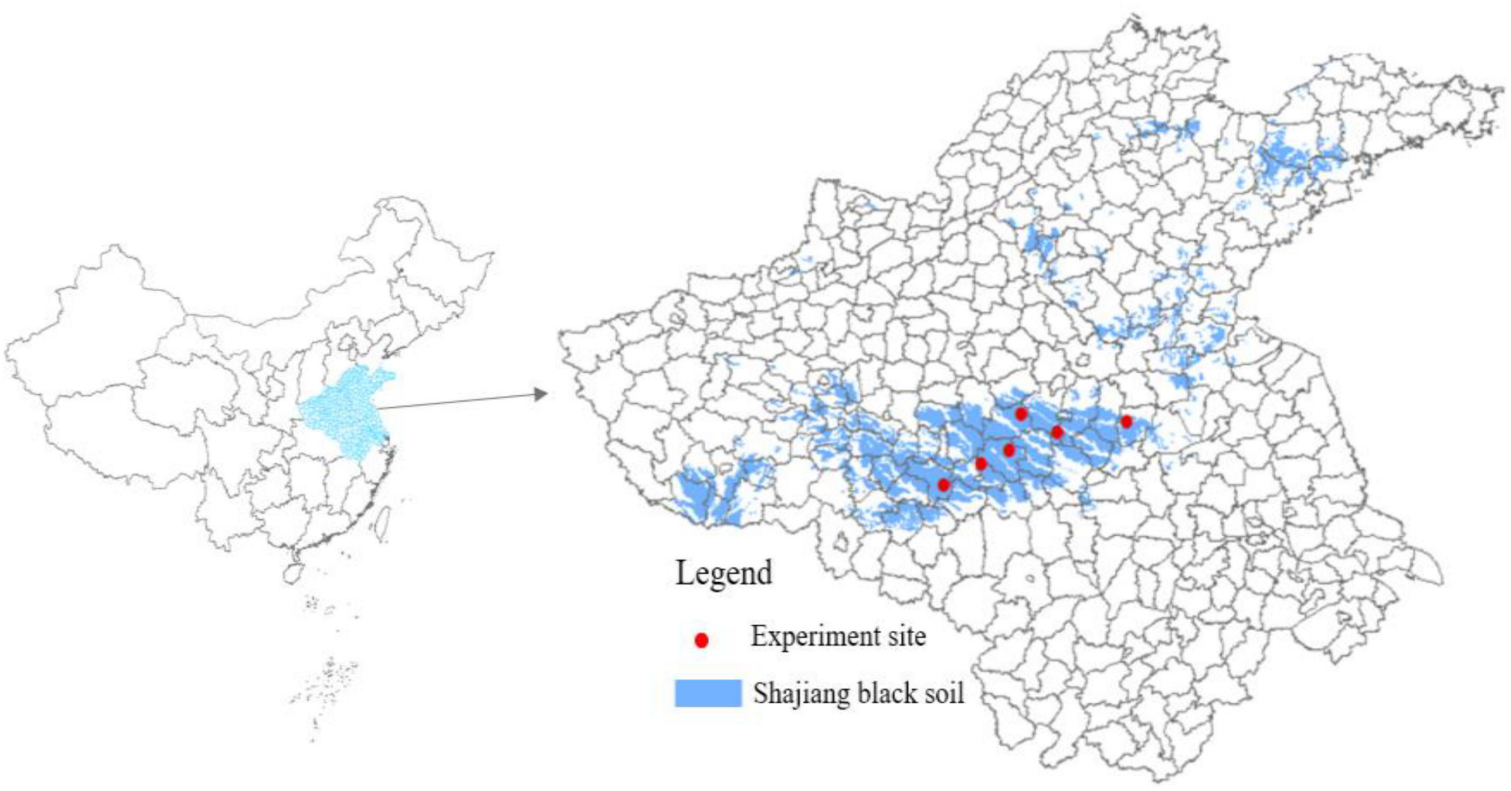
Location of experiment sites included in this study. The distribution of Shajiang black soil is shown in blue, based on data from the Soil Science Database (http://vdb3.soil.csdb.cn/). The experimental sites, arranged from west to east, are located in the following cities: Funan (FN), Yongxing (YX), Kantong (KT), Guoyang (GY), Mengcheng (MC), and Wuhe (WH).

A six-site network experiment was established in October 2021 in the Shajiang Black Soil (Vertisol) region of the North China Plain (**Figure 1**). The experimental sites were located in Funan (FN), Yongxing (YX), Kantong (KT), Guoyang (GY), Mengcheng (MC), and Wuhe (WH), all within the central distribution area of Shajiang Black Soil. To reduce experimental heterogeneity, all sites were selected on level terrain. The region has a warm temperate monsoon climate, with mean annual temperatures ranging from 14.9 to 16.5 °C. Mean annual precipitation varied across sites from 823.9 to 985.2 mm. All sites were rain-fed, with no irrigation applied. Although all sites were classified as Vertisols (Staff, 2010), their initial soil physicochemical properties and textures differed substantially. Detailed site-specific characteristics are provided in **Table 1**.

**Table 1:** Site descriptions and initial soil properties of 0–20 cm layer.

Climate and initial soil properties	Funan (FN)	Yongxing (YX)	Kantong (KT)	Guoyang (GY)	Mengcheng (MC)	Wuhe (WH)
Latitude	33°45′N	33°21′N	33°27′N	33°54′N	32°40′N	34°01′N
Longitude	116°34′E	117°08′E	116°53′E	117°24′E	115°42′E	118°27′E
Elevation, m	20	29	27	29.5	21	16
Mean annual air temperature, °C	15.2	15.2	14.9	15.1	16.5	15.7
Mean annual precipitation, mm	985.2	823.9	826	851.6	872	896
pH(H_2_O, 1:2.5)	5.7	5.07	5.2	5.6	7.5	7.4
SOM, g kg^-1^	18.3	23.6	16.3	21.8	19.4	19.2
Bulk density, g cm^-3^	1.26	1.25	1.31	1.35	1.34	1.29
Available N, mg kg^-1^	63.26	106.67	83.51	111.35	119.33	78.8
Available P, mg kg^-1^	30.55	42.97	23.61	40.68	34.23	18.21
Available K, mg kg^-1^	140.78	117.76	116.17	108.19	100.06	99.66
Sand (2000-50 μm), %	18.5	54.7	52.1	10.4	8.2	64.7
Silt (50-2 μm),%	45.4	15.9	16.3	56.8	54.1	18.9
Clay (<2 μm), %	36.1	29.4	31.6	32.8	37.7	16.4
Soil texture	Clay loam	Sandy clay loam	Sandy clay loam	Silty clay loam	Silty clay loam	Sandy loam

### 2.2 Experimental design and field operation

The experiment was conducted using a randomized complete block design with three replicates and four treatments: no fertilizer (Control), pure chemical fertilizer (NPK), and chemical fertilizer partially substituted with 15% (15%M) or 30% (30%M) pig manure. The control treatment received no fertilizer inputs-neither chemical nor organic-after the experiment began, although all sites had previously followed conventional local fertilization practices. For the NPK treatment, application rates were calculated based on initial soil fertility, crop nutrient demand, and nutrient balance using baseline data (**Table 1**). To ensure consistency, an average rate derived from all six sites was applied uniformly.

In the 15%M and 30%M treatments, 15% and 30% of the chemical nitrogen (N) supplied by pig manure, following the principle of nitrogen equivalence. All fertilizer inputs were standardized and reported as pure N, P_2_O_5_, K_2_O, and pig manure amounts (**Table 2**). Each year, the composted pig manure applied to wheat was identical to that applied to maize and was sourced from the same farm to ensure consistency in manure quality throughout the three-year experiment. The organic carbon and total nitrogen contents of the composted pig manure were 19.9% and 1.4% in 2021, 23.9% and 2.1% in 2022, and 21.1% and 1.7% in 2023, respectively. Manure application rates were based on dry weight, with a consistent moisture content of approximately 12% each year.

**Table 2:** Specific fertilizer application amount under each treatment.

Treatment	Description	Wheat season (kg ha^-1^)				Maize season (kg ha^-1^)			
		Pig manure	N	P_2_O_5_	K_2_O	Pig manure	N	P_2_O_5_	K_2_O
Control	No fertilizer	0	0	0	0	0	0	0	0
NPK	Pure chemical fertilizer	0	225	90	75	0	225	75	90
15%M	Chemical fertilizer was partially substituted by 15% manure	1950	191	52	42	1950	191	42	52
30%M	Chemical fertilizer was partially substituted by 30% manure	3900	157	25	20	3900	157	20	25

Plot sizes were at least 160 m² (20 m × 8 m), with larger dimensions where required for machinery access. After maize harvest, soils were rotary-tilled before wheat sowing in October, whereas maize was directly sown following wheat harvest in June. Maize was planted at a density of 0.25 m × 0.6 m (intra- and inter-row spacing, respectively). All organic fertilizer, phosphorus, potassium, and 60% of nitrogen were applied as basal dressing, while the remaining 40% of nitrogen was top-dressed at the jointing stage of wheat and the large-flower-bud stage of maize in each season.

### 2.3 Soil and crop sampling and analysis

During crop maturity (2021 to 2023), undisturbed soil cores (5.0 cm diameter and 5.0 cm height) were collected from each plot at 7.5–12.5 cm depth and oven-dried at 105 °C to determine bulk density (BD), representing the 0–20 cm soil layer. Composite soil samples (~1 kg) were collected from the 0–20 cm layer using a five-point sampling method. Soil properties: pH was measured in a 1:5 soil-to-water suspension. Soil organic matter (SOM) was determined using the H_2_SO_4_–K_2_Cr_2_O_7_ oxidation method. Total nitrogen (TN) and available nitrogen (A.N) were measured using a Kjeldahl nitrogen analyzer with standard and semi-micro digestion methods. Total phosphorus (TP) was determined by HClO_4_; digestion and molybdate colorimetry, while available phosphorus (A.P) was extracted with 0.5 M NaHCO_3_ and measured by UV spectrophotometer. Total potassium (TK) and available potassium (A.K) were analyzed using flame photometry. Soil texture was determined by laser diffraction particle size analyzer. All analytical procedures followed (Lu, 1999). Elemental stoichiometric: Elemental ratios (C:N, C:P, C:K, N:P, N:K, and P:K) were calculated as molar ratios (Liu et al., 2017). Maize yield was determined from the central 10 m × 1.2 m area (two rows) of each plot, standardized to 14% grain moisture (Jiang et al., 2025). Wheat yield was assessed from a 1 m × 1 m quadrat per plot and adjusted to 13% grain moisture.

### 2.4 Soil quality index and sustainability yield index evaluation

Soil quality was assessed using the soil quality index (SQI), which was derived from a minimum data set (MDS) method (Rinot et al., 2019). All indicators were first standardized, after which the MDS was identified using principal component analysis (PCA) combined with correlation analysis of nine soil variables. Five principal components (PCs) with eigenvalues greater than 1 were retained (**Table 3**), collectively accounting for 92.55% of the total variance. Within each PC, variables with absolute loadings within 10% of the maximum and ≥0.5 were selected. Where multiple indicators were retained within a single PC, those with lower weights and correlation coefficients exceeding 0.6 were excluded to avoid redundancy (Li et al., 2021a). Consequently, SOM, pH, BD, TP, and a.K were selected for inclusion in the MDS (**Table 3**).

**Table 3:** Load matrix and indicators included in the Minimum dataset.

Component	Variables	Principal components PCs					Included
		PC-1	PC-2	PC-3	PC-4	PC-5	
1	SOM	0.841	-0.020	0.208	-0.240	0.431	Yes
2	a.P	0.817	-0.165	0.133	0.320	-0.170	
3	TN	0.803	0.252	0.273	-0.017	0.240	
4	a.N	0.707	0.461	0.278	-0.200	-0.262	
5	pH	-0.424	0.828	-0.018	-0.113	0.201	Yes
6	BD	-0.243	-0.297	0.754	0.167	0.086	Yes
7	TP	0.456	0.375	-0.190	0.711	-0.139	Yes
8	a.K	0.266	-0.461	-0.661	0.241	0.658	Yes
9	TK	0.336	-0.808	0.041	-0.288	-0.194	
Eigenvalue		3.391	2.137	1.579	1.075	1.074	
Percentage of variance (%)		33.912	21.367	15.789	10.746	10.738	
Cumulative percentage of variance (%)		33.912	55.278	71.067	81.814	92.551	

Following MDS selection, indicator weights were determined based on the PCA using Equation 1, and all indicators were then normalized to 0 and 1 using non-linear scoring functions shown in Equation 2 and Equation 3:

(1) $$w_{i}=\sum_{i=1}^{m}\left(\frac{\gamma_{k}}{\sum^{}\gamma }\times a_{kj}\right)$$

(2) $$S_{i}=\;\frac{X_{i}-X_{min}}{X_{max}-X_{min}}$$

(3) $$S_{i}=1-\;\frac{X_{i}-X_{min}}{X_{max}-X_{min}}$$

(4) $$SQI=\sum_{i=1}^{m}w_{i}\times S_{i}$$

Where w_i_ is the weight of the i-th indicator, S_i_ is its normalized score, and m is the number of retained principal components. γ_k_ and a_kj_ represent the eigenvalue and loading of the k-th principal component, respectively. Equation 2 was used when higher values indicated better soil quality; Equation 3 when they indicated poorer quality. The final SQI was computed as the weighted sum of all normalized scores with Equation 4.

Crop yield sustainability index (SYI) was used to evaluate the stability and sustainability of grain yield within the wheat-maize system, which was calculated as Equation 5:

(5) $$SYI=\;\frac{\hat{Y}-\sigma }{Y_{max}}$$

Where Ŷ is the mean yield of wheat or maize, σ is the standard deviation of yield across seasons, and Y_max_ is the maximum observed yield.

### 2.5 Statistical analysis

Data were processed in Excel 2013 and visualized using OriginPro 9.0. ANOVA test was performed in SPSS 19.0. Linear regression assessed relationships among variables. To further quantify the relative importance of soil physical properties, nutrients, soil quality, and ecological stoichiometry for effect on wheat and maize yields, a random-forest approach was used by using the “ randomForest “ packages (Number of trees in each forest = 1000, Number of observations in trees’ terminal note = 2, and Number of mtry features = 4) in R (v3.6.3) software. The Gini coefficient computed from the random-forest structure was calculated to indicate the relative importance (%) of each explanatory variable (Islam et al., 2022).

Structural equation modeling (SEM) was subsequently performed in R using the lavaan package to evaluate the effects of multiple factors on wheat and maize yields and their stability. Six latent variables—namely bulk density (BD), pH, soil organic matter (SOM), nutrient content (total N, P, and K), nutrient availability (available N, P, and K), and nutrient ratios (C:N, C:P, C:K, N:P, N:K, and P:K)—were specified as drivers of soil quality (SQI), which subsequently influenced wheat and maize yields, and ultimately their yield stability (SYI). Prior to modeling, all data were tested for normality and standardized to a mean of 0 and a standard deviation of 1 (Jiang et al., 2024).

## 3 Results

### 3.1 Soil bulk density, pH and SOC as affected by partial organic substitution

**Figure 2:**
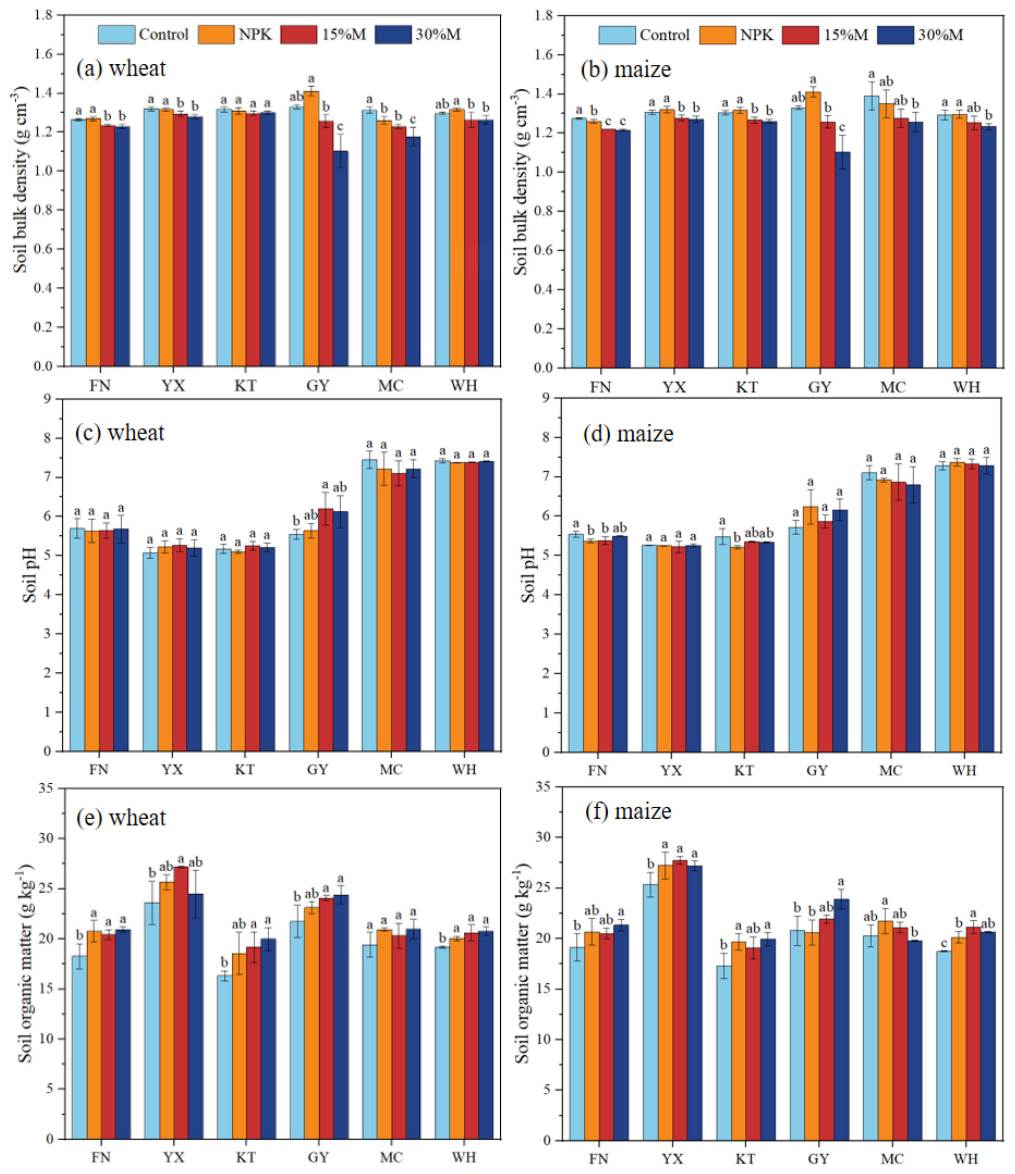
Soil bulk density **(a, b)**, pH **(c, d)**, and organic matter content **(e, f)** as affected by partial organic substitution.

Fertilization significantly affected soil bulk density (BD), with consistent reductions under partial organic substitution relative to both control and NPK treatments. Across sites, BD were significantly declined by 1.5% to 6.3% under 15%M and 1.3% to 17.1% under 30%M, with the strongest decreases at GY and MC (*P* < 0.05; **Figures 2a, b**). Soil pH remained stable across treatments (5.1 to 7.4), within the optimal range for wheat-maize systems (**Figures 2c, d**). SOC increased under all fertilized treatments compared with the control, with greater gains under partial organic substitution (4.6%–22.5%) than NPK (1.5%–13.7%). Relative to NPK, with consistent patterns across wheat and maize seasons Compared to NPK, SOC significantly rose further by 2.8%–5.9% in wheat and 0.77%–8.9% in maize, particularly at KT and GY (*P* < 0.05; **Figures 2e, f**). SOC accumulation was generally higher under the 30%M than 15%M, albeit with greater variability (4.6%–17.5% for 15%M; 3.7%–22.5% for 30%M) (*P* < 0.05; **Figures 2e, f**).

### 3.2 Soil total and available nutrients as affected by partial organic substitution

**Figure 3:**
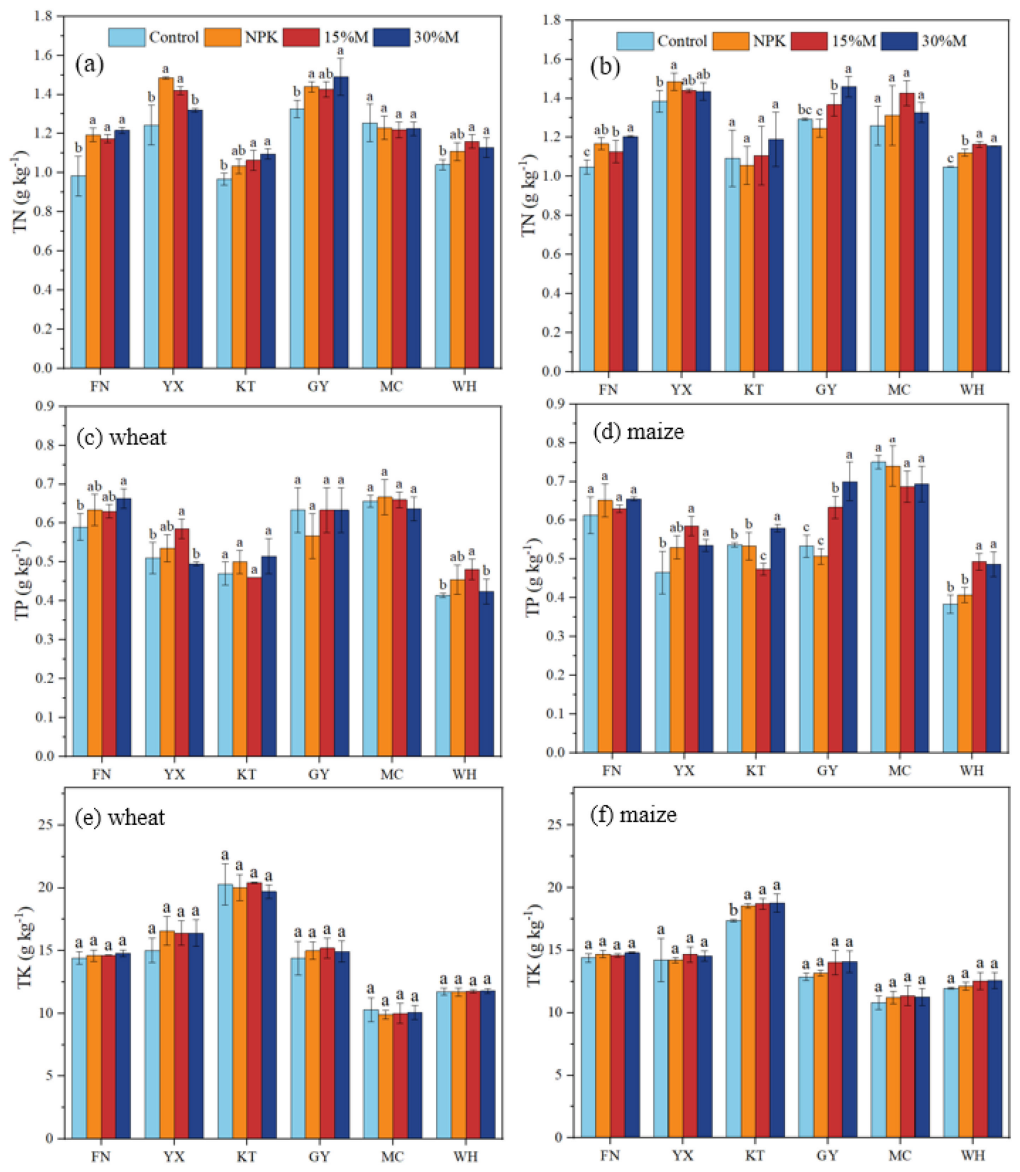
Soil total nitrogen **(a, b)**, phosphorus **(c, d)**, and potassium **(e, f)** as affected by partial organic substitution.

**Figure 4:**
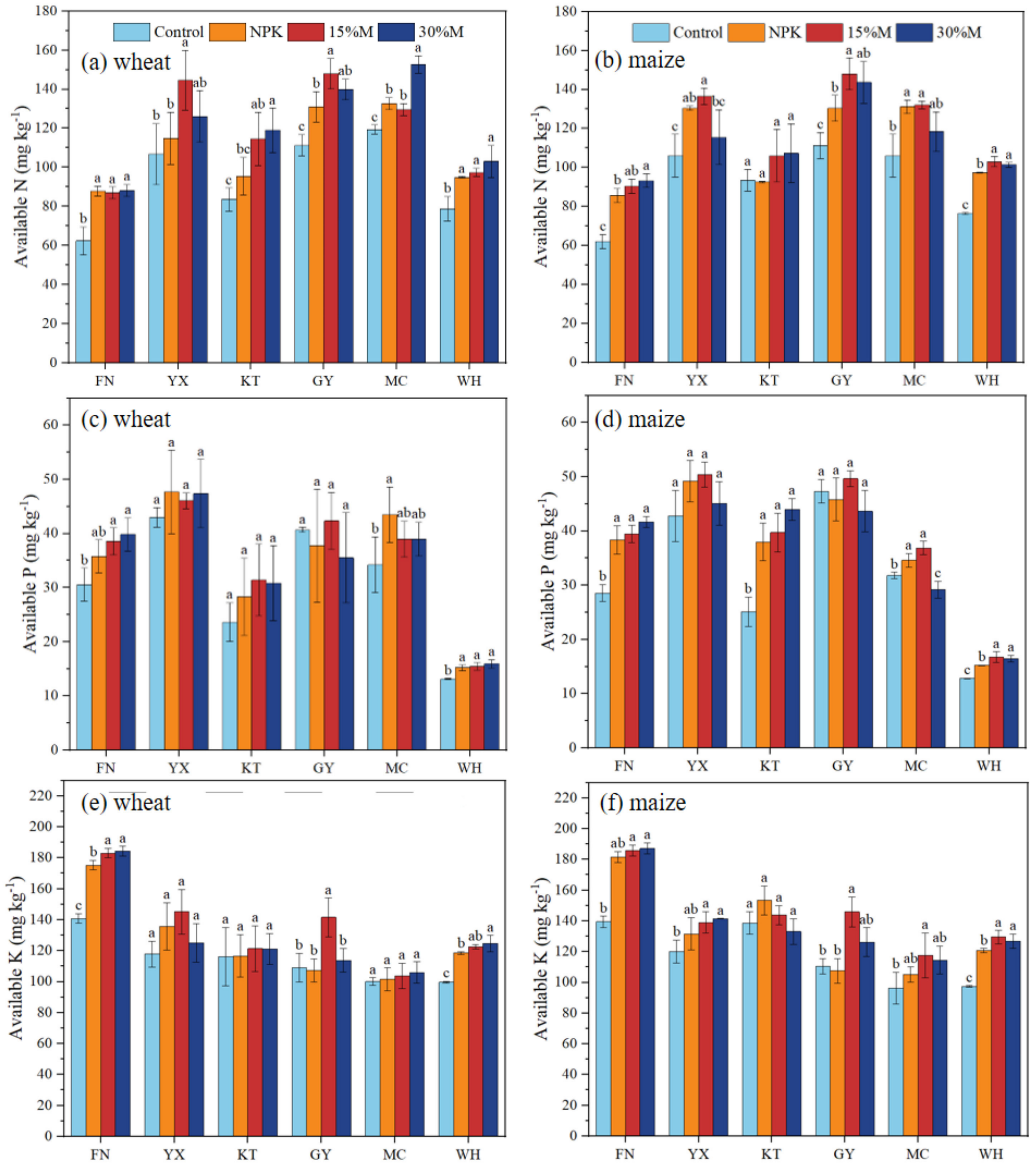
Soil available nitrogen **(a, b)**, phosphorus **(c, d)**, and potassium **(e, f)** as affected by partial organic substitution.

Fertilizer addition increased soil total and available nutrients compared with control, though effects differed by nutrient type, substitution rate, and site (**Figures 3**, **4**). Furthermore, nutrient concentrations were often higher under the 15%M treatment than under 30%M (**Figures 3**, **4**). Total N and P rose by 6.1%–23.7% in wheat and 3.6%–14.9% in maize, with the highest values under NPK, followed by 15%M and then 30%M (*P* < 0.05);. Total K showed no significant treatment effect. Available nutrients responded more strongly than total pools. Available N rose by 7.7%–42.5% in wheat and 0.9%–50.2% in maize, while available P and K increased by 7.1%–45.9% and 0.4%–33.2%, respectively (*P* < 0.05). Across sites, 15%M generally outperformed 30%M for available nutrient supply, particularly at YX, KT, and GY, whereas 30%M occasionally fell below NPK, suggesting potential nutrient constraints at higher substitution levels (**Figure 4**).

### 3.3 Soil elemental stoichiometry as affected by partial organic substitution

Elemental ratios were markedly influenced by fertilization. Under the control, C:N ratios were lowest (8.98–9.81) at MC, GY, and KT, but increased under fertilized treatments to 10 to 12, whereas sites with already high C:N (FN, YX, WH) showed slight declines (**Table 4**). Compared with the control, C:N ratios increased under NPK, 15%M, and 30%M in KT, GY, and MC, where baseline ratios were low, but decreased in FN, YX, and WH, where initial ratios were relatively high (**Table 4**). C:P and N:P ratios indicated excessive P availability (17.2–29.8 and 1.66–3.00 respectively). Both NPK and organic substitution increased these ratios, improving stoichiometric balance at most sites. Similarly, C:K, N:K, and P:K ratios rose under fertilization, with stronger effects under organic substitution-particularly 30%M. However, declines under 30%M at YX suggested localized nutrient imbalance.

**Table 4:** Soil nutrient elemental stoichiometry as affected by partial organic substitution.

Site	Treatment	Wheat						Maize					
		C:N	C:P	C:K	N:P	N:K	P:K	C:N	C:P	C:K	N:P	N:K	P:K
FN	Control	10.8 ± 0.36a	17.9 ± 0.17a	0.74 ± 0.06b	1.66 ± 0.07b	0.068 ± 0.01b	0.041 ± 0.01a	10.6 ± 0.43a	18.1 ± 0.24b	0.77 ± 0.04b	1.71 ± 0.08a	0.073 ± 0b	0.043 ± 0.01a
	NPK	10.1 ± 0.25b	19.1 ± 1.47a	0.82 ± 0.04a	1.89 ± 0.11a	0.082 ± 0a	0.043 ± 0a	10.3 ± 0.38a	18.4 ± 0.15ab	0.82 ± 0.04ab	1.79 ± 0.07a	0.079 ± 0a	0.044 ± 0a
	15%M	10.1 ± 0.19b	18.8 ± 0.07a	0.81 ± 0.02a	1.86 ± 0.04a	0.080 ± 0a	0.043 ± 0a	10.6 ± 0.52a	18.9 ± 0.32a	0.82 ± 0.03ab	1.79 ± 0.07a	0.077 ± 0.01a	0.043 ± 0.01a
	30%M	10.0 ± 0.07b	18.3 ± 0.48a	0.82 ± 0.01a	1.84 ± 0.06a	0.082 ± 0a	0.045 ± 0a	10.3 ± 0.31a	18.9 ± 0.34a	0.84 ± 0.02a	1.84 ± 0.02a	0.081 ± 0a	0.044 ± 0a
YX	Control	11.0 ± 0.57a	26.8 ± 0.33a	0.92 ± 0.14a	2.44 ± 0.11b	0.083 ± 0.01a	0.034 ± 0.01a	10.6 ± 0.94a	32 ± 5.35a	0.85 ± 0.05a	3 ± 0.24ab	0.08 ± 0ab	0.027 ± 0b
	NPK	10.0 ± 0.32a	27.9 ± 1.03a	0.90 ± 0.04a	2.78 ± 0.19a	0.090 ± 0.01a	0.032 ± 0a	10.7 ± 0.84a	29.8 ± 0.23a	0.85 ± 0.05a	2.81 ± 0.24a	0.08 ± 0a	0.029 ± 0.01ab
	15%M	11.1 ± 0.13a	26.9 ± 1.09a	0.96 ± 0.06a	2.43 ± 0.07b	0.087 ± 0.01a	0.036 ± 0.01a	11.2 ± 0.23a	27.5 ± 1.55a	0.86 ± 0.03a	2.46 ± 0.09ab	0.077 ± 1ab	0.031 ± 0a
	30%M	10.8 ± 0.96a	28.7 ± 3.06a	0.86 ± 0.03a	2.67 ± 0.05a	0.081 ± 0a	0.03 ± 0a	11.0 ± 0.54a	29.5 ± 0.31a	0.84 ± 0.05a	2.69 ± 0.16b	0.076 ± 0b	0.029 ± 0ab
KT	Control	9.81 ± 0.41a	20.2 ± 1.54b	0.47 ± 0.03b	2.07 ± 0.20a	0.048 ± 0b	0.023 ± 0a	9.21 ± 0.51b	18.7 ± 1.57c	0.71 ± 0.03c	2.04 ± 0.29a	0.077 ± 0a	0.038 ± 0b
	NPK	10.4 ± 1.46a	21.5 ± 1.6ab	0.54 ± 0.06ab	2.07 ± 0.16a	0.052 ± 0ab	0.025 ± 0a	10.8 ± 0.53a	21.5 ± 2.23ab	0.8 ± 0.02a	1.99 ± 0.31a	0.074 ± 0.01a	0.038 ± 0b
	15%M	10.5 ± 0.43a	24.2 ± 1.97a	0.54 ± 0.05ab	2.31 ± 0.11a	0.052 ± 0ab	0.023 ± 0a	10.1 ± 0.74ab	23.4 ± 0.65a	0.75 ± 0.01b	2.33 ± 0.24a	0.075 ± 0.01a	0.032 ± 0.01a
	30%M	10.6 ± 0.83a	22.6 ± 0.91ab	0.59 ± 0.05a	2.14 ± 0.22a	0.056 ± 0a	0.026 ± 0a	9.79 ± 0.92ab	20 ± 0.35bc	0.8 ± 0.02ab	2.05 ± 0.21a	0.082 ± 0.01a	0.04 ± 0b
GY	Control	9.50 ± 0.53a	20.1 ± 3.13a	0.88 ± 0.02a	2.11 ± 0.25b	0.093 ± 0.01a	0.044 ± 0.01a	9.32 ± 0.61a	22.6 ± 1.41ab	0.94 ± 0.04a	2.43 ± 0.13ab	0.101 ± 0ab	0.041 ± 0bc
	NPK	9.32 ± 0.34a	23.9 ± 2.77a	0.89 ± 0.02a	2.56 ± 0.23a	0.096 ± 0.01a	0.038 ± 0a	9.6 ± 0.22a	23.6 ± 2.21a	0.91 ± 0.07a	2.46 ± 0.18a	0.095 ± 0.01b	0.038 ± 0.01c
	15%M	9.78 ± 0.35a	22.2 ± 2.11a	0.92 ± 0.04a	2.26 ± 0.14ab	0.094 ± 0.01a	0.042 ± 0.01a	9.3 ± 0.57a	20.1 ± 0.82b	0.91 ± 0.08a	2.16 ± 0.17bc	0.098 ± 0ab	0.045 ± 0ab
	30%M	9.52 ± 0.82a	22.5 ± 2.64a	0.95 ± 0.07a	2.36 ± 0.1ab	0.100 ± 0.01a	0.043 ± 0.01a	9.5 ± 0.75a	19.9 ± 2.21b	0.99 ± 0.1a	2.09 ± 0.09c	0.104 ± 0a	0.05 ± 0a
MC	Control	8.98 ± 0.35b	17.2 ± 1.49a	1.10 ± 0.12a	1.91 ± 0.18a	0.122 ± 0.01a	0.064 ± 0.01a	9.4 ± 1.21a	15.7 ± 0.5a	1.09 ± 0.04a	1.68 ± 0.17b	0.117 ± 0.01a	0.07 ± 0a
	NPK	9.87 ± 0.42a	18.3 ± 1.13a	1.23 ± 0.04a	1.85 ± 0.04a	0.124 ± 0a	0.067 ± 0.00a	9.7 ± 1.5a	17.1 ± 2.07a	1.13 ± 0.1a	1.77 ± 0.1ab	0.118 ± 0.02a	0.066 ± 0.01a
	15%M	9.65 ± 0.29a	17.8 ± 0.57a	1.18 ± 0.11a	1.85 ± 0a	0.122 ± 0.01a	0.066 ± 0.01a	8.6 ± 0.58a	17.8 ± 0.63a	1.08 ± 0.1a	2.09 ± 0.22a	0.126 ± 0.01a	0.061 ± 0.01a
	30%M	9.93 ± 0.19a	19.1 ± 1.13a	1.21 ± 0.06a	1.93 ± 0.09a	0.122 ± 0.01a	0.063 ± 0a	8.7 ± 0.35a	16.6 ± 1.1a	1.02 ± 0.07a	1.92 ± 0.17ab	0.118 ± 0a	0.062 ± 0.01a
WH	Control	10.7 ± 0.34a	26.8 ± 0.21a	0.95 ± 0.03b	2.51 ± 0.10a	0.089 ± 0b	0.035 ± 0b	10.4 ± 0.01a	28.4 ± 1.7a	0.91 ± 0.01b	2.74 ± 0.17a	0.088 ± 0a	0.032 ± 0b
	NPK	10.5 ± 0.57a	25.7 ± 2.07a	0.99 ± 0.03ab	2.46 ± 0.29a	0.095 ± 0.01ab	0.039 ± 0.01ab	10.4 ± 0.26a	28.7 ± 1.22a	0.96 ± 0.03ab	2.76 ± 0.17a	0.093 ± 0a	0.034 ± 0b
	15%M	10.3 ± 0.09a	24.9 ± 2.37a	1.02 ± 0.05a	2.42 ± 0.21a	0.099 ± 0a	0.041 ± 0a	10.5 ± 0.18a	24.9 ± 1.07b	0.98 ± 0.03a	2.36 ± 0.09b	0.093 ± 0a	0.039 ± 0.01a
	30%M	10.7 ± 0.46a	28.5 ± 1.66a	1.02 ± 0.01a	2.66 ± 0.13a	0.096 ± 0ab	0.036 ± 0b	10.4 ± 0.04a	24.7 ± 1.7b	0.95 ± 0.05ab	2.38 ± 0.15b	0.092 ± 0.01a	0.039 ± 0a

Different lowercase letters within the same column indicate significant differences between treatments at p < 0.05.

### 3.4 Soil quality and crop yield as affected by partial organic substitution

**Figure 5:**
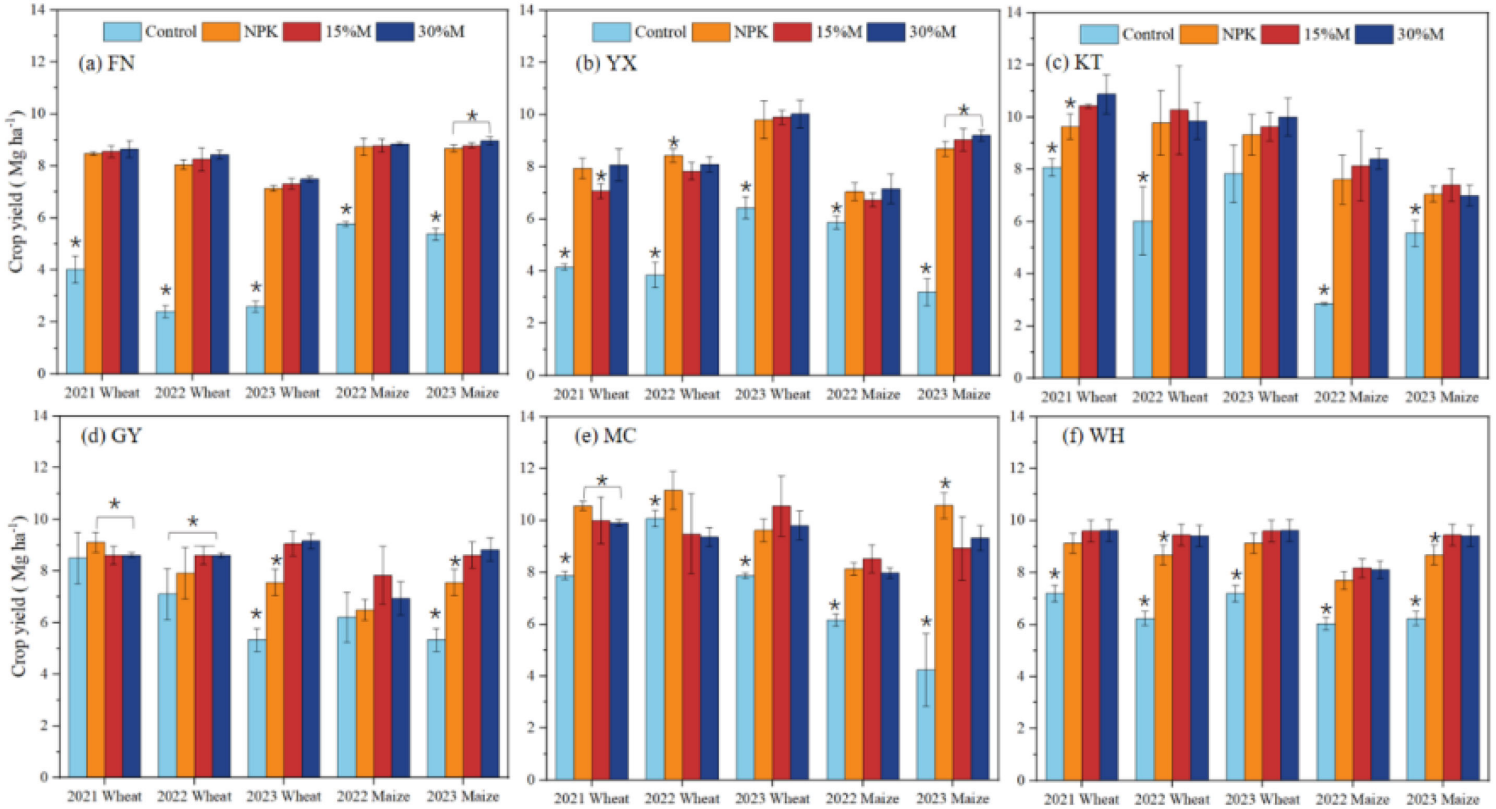
Crop yield of wheat and maize as affected by partial organic substitution. The six experimental sites **(a–f)** comprised Funan (FN), Yongxing (YX), Kantong (KT), Guoyang (GY), Mengcheng (MC), and Wuhe (WH).

Soil quality index (SQI) increased markedly under fertilization, with the greatest improvements under partial substitution (**Figure 5**; **Table 5**). Relative to the control, SQI significantly rose by 230%–249% under 15%M and 278%–416% under 30%M, exceeding the gains from NPK (136%–198%) (*P* < 0.05). Enhancements were greater in maize than wheat, following the order: 30%M > 15%M > NPK. Crop yields were extremely low under the control (2.4–2.5 Mg ha^−1^ for wheat, 2.8–3.2 Mg ha^−1^ for maize). All fertilized treatments significantly improved yields, with partial substitution frequently outperforming NPK. Wheat and maize yields increased by up to 251% and 194%, respectively, with site-specific differences: 30%M was generally superior, except at KT and MC where 15%M yielded better performance. Yield stability, as measured by the sustainability yield index (SYI), improved under all fertilization regimes. On average, SYI rose by 13.9% under 30%M, 9.4% under NPK, and 8.9% under 15%M (*P* < 0.05).

**Table 5:** Soil quality index (SQI) and yield sustainability index (SYI) as affected by partial organic substitution.

Site	Treatment	SQI					SYI				
		Wheat			Maize		Wheat			Maize	
		2021	2022	2023	2022	2023	2021	2022	2023	2022	2023
FN	Control	0.125	0.196	0.127	0.126	0.124	0.797	0.965	0.814	0.916	0.848
	NPK	0.113	0.114	0.146	0.119	0.272	0.984	0.939	0.956	0.971	0.971
	15%M	0.125	0.130	0.196	0.190	0.163	0.950	0.943	0.906	0.981	0.946
	30%M	0.390	0.397	0.383	0.383	0.401	0.925	0.989	0.956	0.964	0.974
YX	Control	0.131	0.191	0.137	0.190	0.105	0.946	0.928	0.773	0.712	0.869
	NPK	0.257	0.206	0.211	0.233	0.282	0.901	0.900	0.940	0.938	0.870
	15%M	0.252	0.257	0.211	0.345	0.415	0.923	0.918	0.917	0.904	0.944
	30%M	0.322	0.458	0.446	0.400	0.418	0.862	0.860	0.929	0.960	0.904
KT	Control	0.125	0.158	0.144	0.135	0.127	0.822	0.875	0.685	0.829	0.748
	NPK	0.399	0.376	0.387	0.365	0.338	0.904	0.770	0.809	0.929	0.838
	15%M	0.520	0.619	0.633	0.623	0.672	0.986	0.706	0.760	0.871	0.903
	30%M	0.701	0.794	0.652	0.659	0.552	0.871	0.909	0.858	0.899	0.859
GY	Control	0.141	0.125	0.193	0.144	0.137	0.889	0.732	0.747	0.839	0.839
	NPK	0.169	0.171	0.184	0.191	0.168	0.935	0.879	0.784	0.881	0.881
	15%M	0.590	0.582	0.624	0.570	0.622	0.918	0.756	0.917	0.884	0.919
	30%M	0.592	0.539	0.826	0.611	0.755	0.982	0.822	0.982	0.916	0.951
MC	Control	0.149	0.160	0.137	0.123	0.191	0.862	0.824	0.895	0.508	0.976
	NPK	0.671	0.686	0.697	0.640	0.679	0.967	0.947	0.880	0.927	0.909
	15%M	0.621	0.600	0.701	0.629	0.648	0.825	0.877	0.703	0.767	0.789
	30%M	0.776	0.586	0.769	0.759	0.861	0.974	0.950	0.926	0.907	0.895
WH	Control	0.082	0.060	0.061	0.043	0.151	0.829	0.833	0.729	0.859	0.929
	NPK	0.127	0.175	0.154	0.192	0.253	0.729	0.925	0.889	0.789	0.893
	15%M	0.199	0.172	0.193	0.271	0.377	0.949	0.969	0.956	0.948	0.979
	30%M	0.197	0.212	0.181	0.363	0.314	0.929	0.928	0.936	0.967	0.977

### 3.5 Correlations and driving mechanisms

**Figure 6:**
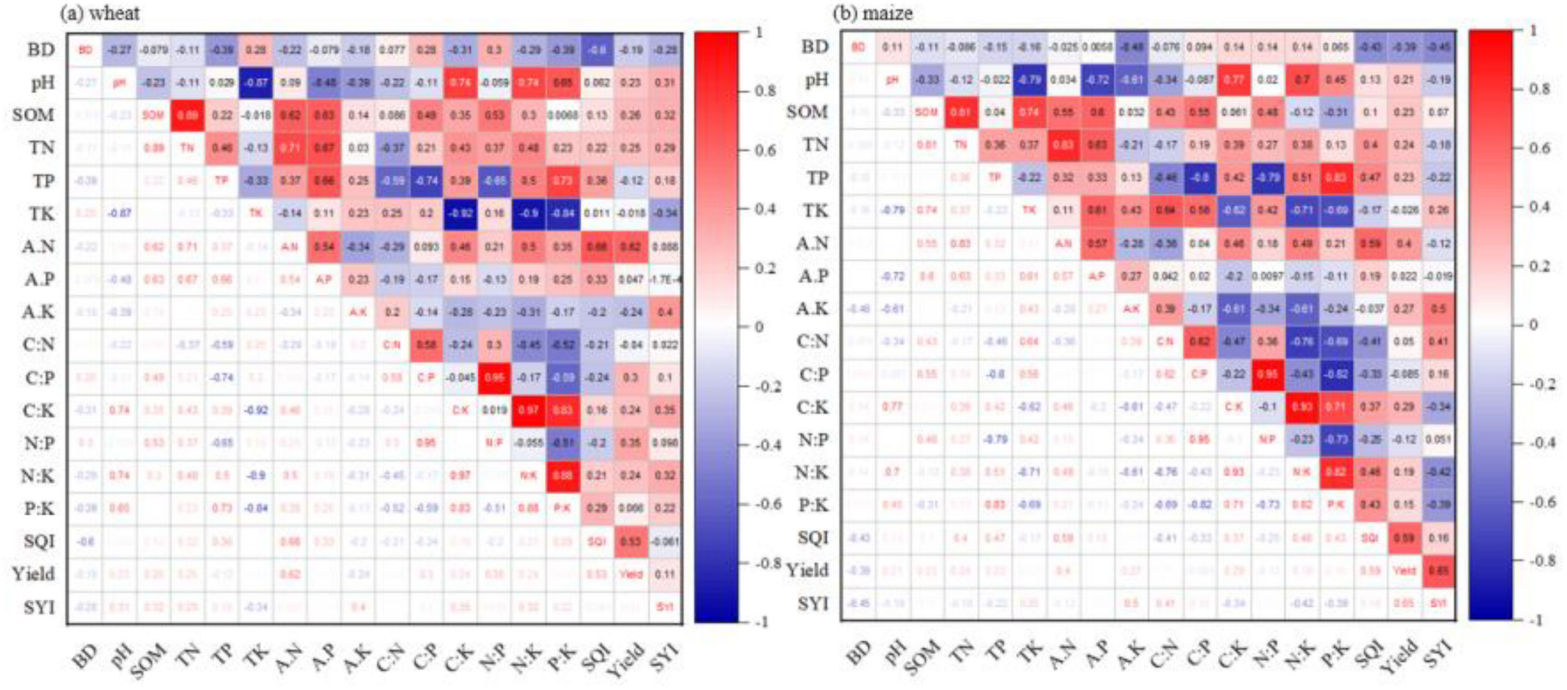
Correlation between crop yield, soil quality, physicochemical properties, and elemental stoichiometry under partial organic substitution in **(a)** wheat and **(b)** maize system. BD, bulk density; pH; SOM, soil organic matter; TN, total nitrogen; TP, total phosphorus; TK, total potassium; A.N, available nitrogen; A.K, available potassium; C:N ratio, C:P ratio, C:K ratio, N:P ratio, N:K ratio, P:K ratio; SQI, soil quality index; and the SYI, crop yield sustainability index.

**Figure 7:**
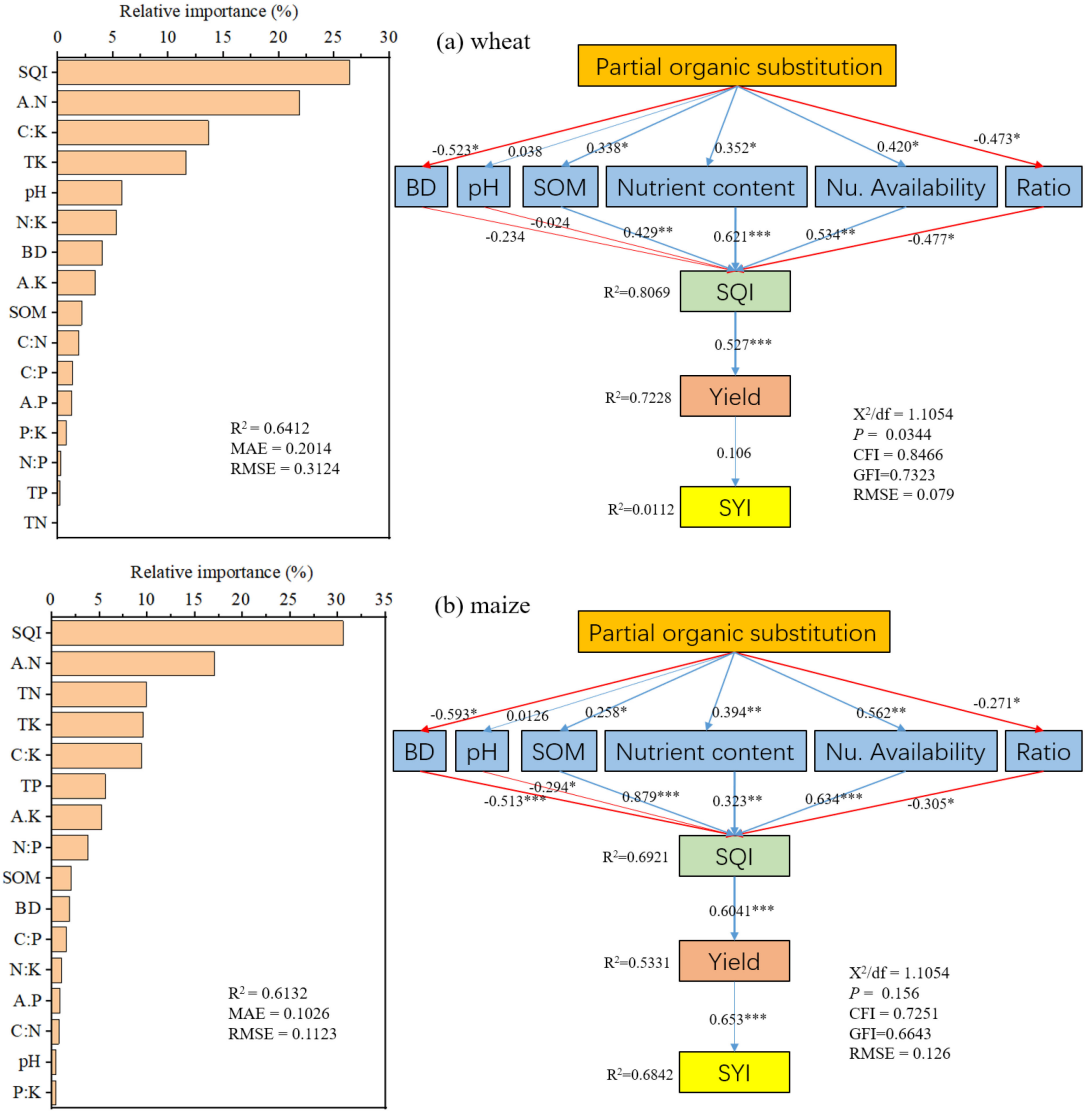
Relative importance **(a)** and structural equation model (SEM) contributions **(b)** of different influencing factors on wheat **(a)** and maize **(b)** yield andyield stability under partial organic substitution. BD, bulk density; pH; SOM, soil organic matter; TN, total nitrogen; TP, total phosphorus; TK, total potassium; A.N, available nitrogen; A.K, available potassium; C:N ratio, C:P ratio, C:K ratio, N:P ratio, N:K ratio, P:K ratio; SQI, soil quality index; and the SYI, crop yield sustainability index.

Correlation analysis revealed strong negative effects of BD on SQI (r = –0.60 in wheat, –0.43 in maize). SOC correlated positively with TN, available N and P, and C:P and N:P ratios (r = 0.46–0.89). Soil pH was strongly associated with K availability and K-related stoichiometry, while total nutrient contents correlated closely with their available forms (**Figure 6**). Random forest models identified SQI as the dominant predictor of yield (26.4% for wheat, 30.5% for maize), followed by available N (17%–22%). Additional contributors included C:K ratio, total K, soil pH, and N:K ratio for wheat, and total N, P, K, and available K for maize (**Figure 7a**). SEM analysis confirmed that partial organic substitution improved elemental ratios, which enhanced SQI and in turn promoted yield and stability. Key pathways involved nutrient availability, total nutrient content, BD reduction, and SOC accumulation, with nutrient-related effects stronger than those of SOC (**Figure 7b**).

## 4 Discussion

### 4.1 Response of soil bulk density, pH, SOC, and nutrients to partial organic fertilizer substitution

Across all six Vertisol sites, partial substitution of chemical fertilizer with organic manure (15%M and 30%M) significantly reduced bulk density, with the strongest effects at GY and MC, where initial compaction and clay content were greatest. These reductions were closely linked to SOC increases, as organic inputs promoted aggregate formation, enhanced pore connectivity, and reduced compaction (Fang et al., 2021). In initially, as fresh organic fertilizers decompose, microorganisms (e.g., fungi and actinomycetes) secrete polysaccharides and mucilage that bind fine soil particles into microaggregates (Wen et al., 2021). As decomposition progresses, stable humic substances (e.g., humic and fulvic acids) form and, together with clay minerals and cations (e.g., Ca^2+^), promote the formation of water-stable macroaggregates (Wen et al., 2021). This hierarchical pore structure enhances soil porosity and reduces bulk density. Similar long-term evidence shows that sustained organic amendments can decrease bulk density by 2.29–13.74% and increase SOC by 15.7–132.7% in Vertisols (Guo et al., 2019), reinforcing the structural benefits of organic inputs in fine textured soils.

Nutrient dynamics also reflected the interactive roles of chemical and organic fertilization. While total N, P, and K contents under partial substitution remained comparable to NPK, available nutrient pools were consistently higher (**Figure 4**). This finding is particularly significant, as more than 4 million hectares of Vertisols in the Huang–Huai–Hai Plain have long experienced severe soil nutrient imbalances, characterized by nutrient depletion, low nutrient availability, slow accumulation of available nutrients, and deficiencies in phosphorus and nitrogen, as well as micronutrients such as zinc, boron, and molybdenum (Xiong et al., 2020; Li et al., 2011). The implementation of an integrated organic–inorganic fertilization strategy represents an effective approach to concurrently ameliorate macronutrient (P and N) limitations and their availability in this region. This enhancement can be attributed to several processes: (i) competition between organic carbon and nutrient ions (NH_4_^+^, PO_4_^3−^, K^+^) for mineral sorption sites, which reduces fixation (Jilling et al., 2021; Xu and Tsang, 2024); (ii) organic acid-mediated mobilization of native nutrients; and (iii) stimulation of microbial activity, enzymatic processes, and nutrient turnover (Wu et al., 2006; Beermann et al., 2015). These mechanisms collectively increase nutrient bioavailability, particularly at YX, KT, and GY where organic substitution showed the greatest effects. Thus, partial substitution enhanced not only soil physical conditions but also nutrient accessibility beyond that achieved by chemical fertilization alone (Singh et al., 2024).

### 4.2 Response of soil elemental stoichiometry to partial organic fertilizer substitution

Stoichiometric ratios provide integrative indicators of soil nutrient balance and plant–microbe interactions (Liu et al., 2017). In this study, C:N ratios were below the optimal 10–12:1 in MC, GY, and KT under unfertilized conditions (Bing et al., 2015), but increased to optimal ranges under fertilization, driven primarily by SOC accumulation. Vertisols, inherently low in nutrients, suffer from a severe shortage of exogenous organic inputs in the absence of organic fertilizer application (Xiong et al., 2020). Continuous carbon removal through grain and straw harvest exacerbates this deficit, driving the soil carbon cycle into a persistent imbalance (Wang et al., 2021). Under these conditions, soil microorganisms accelerate the decomposition of the limited soil organic matter (SOM) to obtain energy (Huang et al., 2018). However, the lack of fresh carbon inputs restricts replenishment of the soil organic carbon (SOC) pool, leading to slow accumulation or continual decline (Huang et al., 2018). Meanwhile, microbial decomposition of SOM releases mineral nitrogen, resulting in rapid carbon depletion coupled with relative nitrogen accumulation and maintaining a low soil C:N ratio (typically below 10:1; **Table 3**). In contrast, manure application supplies substantial amounts of organic carbon and nitrogen, stimulating microbial activity—particularly bacterial proliferation—and promoting SOC accumulation (Wu et al., 2006). The increase in SOC arises both from the carbon contained in the manure and from microbial residues formed during decomposition (Zhang et al., 2024), thereby enhancing SOC stability and restoring the soil C:N ratio to an optimal range.

Meanwhile, C:P and N:P ratios revealed a regional excess of phosphorus, consistent with surveys across the North China Plain (Xiong et al., 2021). Both chemical and organic fertilization increased these ratios by elevating soil C and N while P remained relatively stable, thereby alleviating P-induced stoichiometric imbalance. This finding provides a cost-effective strategy for medium- to low-yield lime concretion Vertisols through organic fertilizer substitution aimed at “increasing carbon and supplementing nitrogen.” By enhancing soil C and N pools, this approach corrects phosphorus-driven imbalances without resorting to costly phosphorus-reduction measures, optimizing C:P and N:P ratios while ensuring adequate nutrient availability for crops (**Table 4**). Moreover, it mitigates the risk of phosphorus runoff and leaching, simultaneously improving soil fertility and protecting the environment.

Ratios involving K (C:K, N:K, P:K) were generally lower than crop uptake requirements, suggesting limitations of C, N, and P relative to high background K (Li et al., 2022). Partial organic substitution, especially 30%M, improved these ratios more effectively than NPK, though imbalances persisted at some sites (e.g., declines at YX). Given that K is less incorporated into microbial biomass yet critical for crop uptake (Wang et al., 2024), maintaining balanced stoichiometry through combined organic-inorganic fertilization is essential for long-term sustainability in Vertisols (Chen et al., 2020).

### 4.3 Response of crop yield and soil quality to partial organic fertilizer substitution

Crop yield and soil quality responded strongly to fertilization, with partial organic substitution outperforming chemical fertilizer alone. Improvements in the Soil Quality Index (SQI) under 15%M and 30%M corresponded to significant yield gains, particularly in maize, reflecting the close link between soil quality and productivity in this system (**Figure 5**, **Table 5**). This is consistent with (Engels, 1993) who reported that maize, as a C4 crop, undergoes a pronounced “explosive growth phase” from jointing to filling, during which nitrogen demand accounts for over 70% of total growth. The gradual nitrogen release from organic fertilizer mineralization aligns with this peak demand. In contrast, wheat, as an overwintering crop, experiences markedly reduced mineralization rates during its reviving stage due to low temperatures, creating a mismatch between nutrient supply and demand; consequently, wheat relies more heavily on chemical fertilizers to meet its nitrogen requirements during this critical period (Page et al., 1978).

Random forest analysis identified SQI as the dominant predictor of yield (26–31%), followed by available N, emphasizing the importance of soil quality. Structural equation modeling indicated that yield gains were mediated through improvements in stoichiometry, nutrient availability, bulk density, and SOC, with nutrient pathways exerting the strongest influence (**Figures 6**, **7**). These results align with previous studies showing that integrated nutrient management enhances both soil function and crop performance more effectively than sole chemical fertilization (Chen et al., 2020; Singh et al., 2024).

Importantly, the observed site-specific variation—for example, stronger benefits for bulk density at GY and MC, or superior yield performance of 15%M at KT and MC—highlights the need to tailor substitution rates to local soil constraints. Overall, a moderate organic substitution rate (~30%) appears effective in improving soil structure, nutrient availability, and crop yield in Vertisols. Nevertheless, site-specific conditions such as compaction and organic matter content should guide local adjustment of substitution rates. Future practices should adopt tailored organic–inorganic strategies to sustain soil fertility and crop productivity.

## 5 Conclusions

This three-year, six-site study shows that partial substitution of chemical fertilizer with organic manure (15–30%) effectively improves soil quality, elemental stoichiometry, and crop productivity in Vertisols of the North China Plain. Organic inputs reduced compaction, increased SOC, enhanced nutrient availability, and corrected stoichiometric imbalances (C:N, C:K, N:K, P:K), leading to higher SQI, improved wheat and maize yields, and greater yield stability, with 30% substitution generally most beneficial. By addressing the key physical, chemical, and biological constraints of Vertisols, partial organic substitution provides a practical pathway for sustainable intensification, highlighting the wider potential of integrated organic–inorganic fertilization to balance productivity and soil health in intensive agroecosystems.

## Data Availability

The original contributions presented in the study are included in the article/supplementary material. Further inquiries can be directed to the corresponding author.
